# A randomised controlled trial of the impact of structured written and verbal advice by community pharmacists on improving hypertension education and control in patients with high blood pressure

**DOI:** 10.1007/s00228-018-2519-0

**Published:** 2018-07-18

**Authors:** Ejaz Cheema, Paul Sutcliffe, Martin O. Weickert, Donald R. J. Singer

**Affiliations:** 1 Warwick Medical School, Gibbet Hill Campus, University of Warwick, Coventry, CV4 7AL UK; 2 WISDEM Centre, University Hospitals Coventry and Warwickshire NHS Trust, Coventry, UK; 3 Centre of Applied Biological and Exercise Sciences, Faculty of Health and Life Sciences, Coventry University, Coventry, UK; 4 Fellowship of Postgraduate Medicine, 12 Chandos street, London, UK

**Keywords:** Community pharmacists, Education, Hypertension, Randomised controlled trial

## Abstract

**Purpose:**

This study was aimed to determine whether structured written and verbal education provided to patients by community pharmacists about high blood pressure (BP) and its treatment would be (a) better retained and (b) be associated with improved BP control as compared to patients receiving verbal advice only.

**Methods:**

The study was designed as a randomised controlled trial and was conducted in the West Midlands, UK, between January 2014 and June 2014. The primary outcome measures were differences in systolic and diastolic BP from baseline and retention of information about high BP assessed with a questionnaire at 2-, 4- and 26-week follow-up points.

**Results:**

A total of 64 adults were included in the study. At the week 26 follow-up, compared to participants in the control group, there was a significant improvement in the knowledge of intervention participants about the risks associated with high BP (*p* < 0.001) and awareness about potential adverse effects of the new BP medicine (*p* < 0.001). Similarly, there was a greater and more significant reduction in systolic BP in favour of the intervention group 8 mmHg (95% CI 2.1–13.3 *p* = 0.009) compared to 6 mmHg (95% CI 0.6–11.7 *p* = 0.02) in the control group at the week 4 follow-up. However, this greater effect of an intervention on BP was not sustained at the 26-week follow-up. For diastolic BP, there was no added effect of the intervention.

**Conclusion:**

This randomised controlled trial suggests that although written advice provided by community pharmacists in comparison to verbal advice was more effective in improving knowledge and understanding of patients about hypertension and its treatment, it did not lead to better blood pressure control.

## Introduction

It has been reported that behavioural interventions—patient-centred counselling, self-monitoring of blood pressure (BP) and structured training courses—on BP management lead to better BP control in patients with hypertension as compared to patients receiving ordinary care [1]. Pooled results from this systematic review that included 15 studies involving 4072 patients reported that patient-centred counselling led to a reduction of 11.1 mmHg in systolic and 3.2 mmHg reduction in diastolic BP [1]. The review did not specify if the patient-centred counselling was written or verbal. Similar findings were reported in a cluster randomised controlled study in the USA [2]. Patients receiving multifactorial interventions including written patient education experienced a reduction of 8 mmHg in systolic BP and achieved a better BP control. Lack of adequate knowledge about high BP has been reported as a barrier to medication adherence by hypertensive patients [3].

In the UK, the New Medicines Service (NMS) allows community pharmacists to explain medicine use to patients with long-term medical conditions such as hypertension. Within this scheme, the advice is structured but verbal, with no specific written information provided on drugs or the disease being treated. There is limited evidence to suggest that provision of written medical advice to patients about a disease and its treatment is better retained by patients than verbal information [4] and leads to better clinical outcomes [2]. The study aimed to determine whether the structured information provided to patients verbally and in writing by community pharmacists about high BP will be associated with improved BP control and be better retained by patients.

## Methods

This study was a 6-month multicentre randomised controlled trial (RCT) conducted across four community pharmacies in the West Midlands area of the UK between January 2014 and June 2014. The trial was registered on ClinicalTrials.gov (Identifier NCT01939860). Participants in both groups were required to attend four visits in total over a period of 6 months (at week 0, 2, 4 and 26).

### Study participants and procedures

All participants 18 years or over, male or female and had been started on a BP medication were eligible for the study. Eligible participants were identified by a member of the pharmacy team. Exclusion criteria included patients under 18 years and patients not capable of giving written consent.

The questionnaire drawn from a 12-item questionnaire developed by the National Institutes of Health [5] was aimed to explore participants’ basic knowledge of BP including awareness about the risks associated with high BP and knowledge about the participants’ new BP medicine. Face validity of the questionnaire was undertaken by seeking feedback from a pharmacists advisory group including six pharmacists as well as from an expert hypertension advisory group based in the West Midlands area. Besides, the questionnaire was also piloted on a group of 20 patients that included patients attending the BP Clinic at a large teaching hospital in the West Midlands and patients attending one of the participating pharmacies in Birmingham. Based on the feedback obtained during pilot work, the wording of some of the questions of the questionnaire was edited to make the questions simpler and easier to understand. The final questionnaire had a Flesch-Kincaid reading grade level of 5.8 [6].

Three readings of systolic and diastolic BP were recorded for both intervention and control groups participants during all four visits (weeks 0, 2, 4 and 26). BP was recorded electronically by trained pharmacy staff using a British Hypertension Society (BHS)-approved Omron BP monitor [7]. As per the BHS guidelines, the final two readings of both systolic and diastolic BP were used to calculate the average readings.

### Intervention and comparator (usual care)

Participants in both groups were asked to continue to take their prescribed anti-hypertensive medications during the study. In addition to usual care, participants in the intervention group received individually tailored information sheets containing structured advice on BP and their anti-hypertensive medication that was prepared using National Institute for Health and Care Excellence (NICE) guidance CG 127 [8]. These information sheets were provided by a trained pharmacist during three face to face sessions (week 0, 2 and 4) over a period of 6 months. All participating pharmacists were provided 30-min training by the chief investigator of the study. Besides, training was provided to pharmacy staff on measuring blood pressure. Pharmacists were specifically instructed not to provide any help with answering the questions as the same questionnaire was used during all four visits. However, pharmacists were allowed to assist the participants in understanding the medical terminologies used in the questions when needed. The participants in the control group received a separate information sheet containing information on the NMS in addition to usual care.

### Data management and analysis

The sample size calculation indicated that a sample size of 54 per group completing the study will provide a power of 80% at the 5% level in a two-tailed test to detect a reduction of a size equal to 0.6 standard deviations (SD) in systolic and diastolic BP. The primary outcome of the study was to detect the difference in systolic and diastolic BP from baseline. The secondary outcome was the retention of information about BP assessed with a questionnaire at 2, 4 and 26 weeks follow-up. ANOVA was used to calculate the mean difference in systolic and diastolic BP (in mmHg). Cross tabulation was used to analyse the responses to hypertension knowledge questions.

## Results

**Fig. 1:**
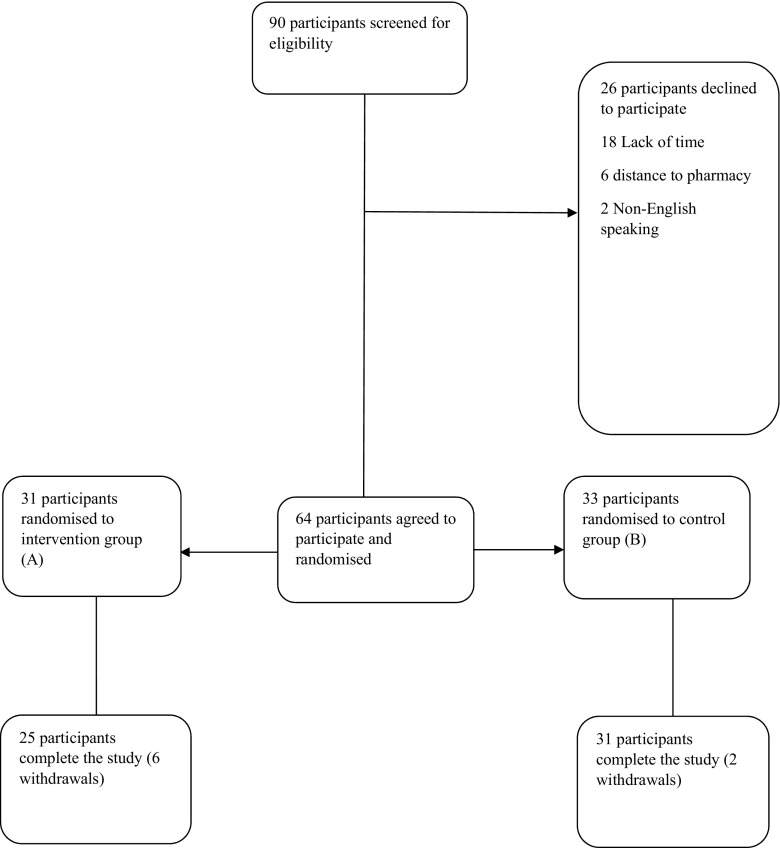
Flow of participants through the study

A total of 64 participants were included in the study (see Fig. 1 for flow of participants through the study). At baseline, no statistically significant differences were found in the demographics of intervention and the control group participants (see Table 1 for participant demographics).

**Table 1:** Participant demographics at Baseline. BMI—body mass index. All data are given in numbers (percentages) unless otherwise indicated

Variables	Intervention	Control
Mean age years (SD)	64.7 (10.5)	60.0 (9.3)
Gender		
Male	14 (45%)	18 (55%)
Female	17 (55%)	15 (45%)
Ethnicity		
White Caucasian	24 (78%)	25 (76%)
South Asian	5 (16%)	6 (18%)
African Caribbean	2 (6%)	2 (6%)
Mean BMI kg/m^2^ (SD)	29.0 (5.9)	30.3 (5.2)
Systolic blood pressure mmHg (SD)	142 (17.0)	143 (16.9)
Diastolic blood pressure mmHg (SD)	79 (11.4)	83 (12.9)
Other medical conditions (self-reported)	6 (19%)	7 (21%)
Diabetes	1(3%)	0
Heart failure	1 (3%)	1 (3%)
Kidney disease	1 (3%)	4 (13%)
Heart attack	1 (3%)	1 (3%)
Stroke	21 (67%)	20 (60%)

### Impact on systolic and diastolic BP

At the end of week 4 follow-up, there was a greater reduction in systolic BP in favour of the intervention group 8 mmHg (95% CI 2.1–13.3 *p* = 0.009) compared to 6 mmHg (95% CI 0.6–11.7 *p* = 0.02) in the control group. However, this greater effect of intervention on systolic BP was not sustained at the 26-week follow-up with little difference remaining between the groups, 8 mmHg (95% CI 2.1–13.3 *p* = 0.01) intervention group vs. 7 mmHg (95% confidence interval 0.6–11.7 *p* = 0.02) control group. For diastolic BP, there was no added effect of the intervention and both groups achieved a similar reduction in diastolic BP at the 26-week follow-up, 4.5 mmHg (95% CI 1.2–7.7 *p* = 0.008) intervention group vs. 5 mmHg (95% CI 1.3–8.8 *p* = 0.009).

### Knowledge about hypertension and its treatment

With regard to the assessment of knowledge about BP, there was a significant improvement in the knowledge of intervention participants in comparison to participants in the control group at the 26-week follow-up including knowledge about the risks associated with high blood pressure (*p* < 0.001), the role of lifestyle measures in reducing high blood pressure (*p* < 0.01) and awareness about potential adverse effects of the new blood pressure medicine (*p* < 0.001) (see Table 2 for percentages of participants correctly answering each hypertension knowledge question).

**Table 2:** Percentages of participants correctly answering each hypertension knowledge question

	Intervention (*n* = 25)	Control (*n* = 31)				Difference between groups expressed by *p* value+		
Knowledge question	Baseline	Follow-up	Difference within group *p* value*	Baseline	Follow-up	Difference within group *p* value*	Baseline	Follow-up
Top blood pressure number should be under 140?	16 (64%)	24 (96%)	*p* = 0.01	18 (58%)	23 (74%)	*p* = 0.25	*p* = 0.65	*p* = 0.02
Lower blood number should be under 90?	15 (60%)	24 (96%)	*p* = 0.02	21 (68%)	24 (77%)	*p* = 0.85	*p* = 0.54	*p* = 0.04
Hypertension is a lifelong disease?	11 (44%)	25 (100%)	*p* < 0.001	20 (65%)	25 (84%)	*p* = 0.29	*p* = 0.12	*p* = 0.02
Hypertension can cause heart attacks?	11 (44%)	25 (100%)	*p* < 0.001	20 (65%)	25 (84%)	*p* = 0.51	*p* = 0.99	*p* = 0.19
Hypertension can cause strokes?	21 (84%)	25 (100%)	*p* = 0.03	26 (84%)	29 (94%)	*p* = 0.09	*p* = 0.47	*p* = 0.36
Hypertension can cause kidney disease?	21 (84%)	25 (100%)	*p* = 0.20	21 (68%)	25 (80%)	*p* = 0.30	*p* = 0.87	*p* = 0.36
Hypertension does not cause asthma?	14 (56%)	23 (92%)	*p* = 0.03	18 (58%)	22 (71%)	*p* = 0.20	*p* = 0.68	*p* = 0.12
Hypertension does not cause cancer?	12 (48%)	19 (76%)	*p* = 0.02	13 (42%)	18 (58%)	*p* = 0.18	*p* = 0.87	*p* = 0.14
Losing weight reduces high blood pressure?	15 (60%)	23 (92%)	*p* = 0.005	12 (39%)	16 (52%)	*p* = 0.39	*p* = 0.04	*p* = 0.41
Cutting salt reduces high blood pressure?	20 (80%)	24 (96%)	*p* = 0.004	26 (84%)	29 (94%)	*p* = 0.44	*p* = 0.70	*p* = 0.25
Cutting alcohol reduces high blood pressure?	19 (76%)	25 (100%)	*p* = 0.18	28 (90%)	30 (97%)	*p* = 0.65	*p* = 0.27	*p* = 0.41
Anti-hypertensive should be taken daily?	19 (76%)	25 (100%)	*p* = 0.002	28 (90%)	30 (97%)	*p* = 0.79	*p* = 0.04	*p* = 0.19
Anti-hypertensive should be taken long-term?	22 (88%)	25 (100%)	*p* = 0.45	28 (90%)	28 (90%)	*p* = 0.34	*p* = 0.27	*p* = 0.11
Name of your new blood pressure medicine?	20 (80%)	25 (100%)	*p* = 0.01	28 (90%)	28 (90%)	*p* = 0.35	*p* = 0.96	*p* = 0.43
Dose of your new blood pressure medicine?	16 (64%)	20 (80%)	*p* = 0.08	20 (65%)	22 (71%)	*p* = 0.81	*p* = 0.81	*p* = 0.25
How your new blood pressure medicine works?	17 (68%)	21 (84%)	*p* = 0.09	22 (71%)	22 (71%)	*p* = 0.03	*p* = 0.07	*p* = 0.01
Awareness about adverse effects?	6 (24%)	21 (84%)	*p* < 0.001	7 (23%)	11 (35%)	*p* = 0.21	*p* = 0.90	*p* < 0.001
Incidence of adverse effects?	10 (40%)	5 (20%)	*p* = 0.12	8 (26%)	6 (19%)	*p* = 0.55	*p* = 0.39	*p* = 1.00

*Chi-square test at *p* < 0.05. *p* value* indicates the difference within the study groups and *p* value+ indicates the difference between the study groups

## Discussion

The findings of this study suggest that although there was a greater reduction in systolic BP in favour of the intervention group compared to control group at the 4-week follow-up, this greater effect of intervention on BP was not sustained at the 26-week follow-up with both groups achieving similar reduction in BP. These findings suggest that verbal advice provided by pharmacists alone was equally effective as the written advice in supporting a reduction in systolic and diastolic BP of hypertensive patients following the introduction of a new BP-lowering medicine. However, compared to verbal advice only, provision of structured verbal and written education was associated with an improvement sustained over 6 months in the knowledge of hypertension.

There can be many possible reasons for the non-sustainability of greater BP control initially achieved by intervention participants over the long-term follow-up. It is not completely clear if the intervention participants initially responded better to their new BP treatment than the control participants. Another reason for the loss of their greater BP control could be that perhaps they needed frequent reminders about high BP and its treatment. A recent study that included 1300 adults with high BP compared two intervention groups who received education about high BP and its treatment through text message reminders and interactive text messaging to a control group receiving standard care. The study reported that those who had received text messages had a slightly greater reduction in their BP and were more likely to have achieved a controlled BP [9].

This study has several limitations. Although, the study reported a greater initial reduction in systolic BP of intervention participants compared to control group, such initial better blood pressure control could have been explained by the risk of confounding factors including the type and dose of antihypertensives used, and degree of patient compliance with their medication. Such confounders could have been addressed by appropriate matching of controls. In addition, we were not able to recruit the initially planned number of participants, resulting in reduced power for assessment of the outcome measures. This was primarily due to the withdrawal of two participating pharmacies from the study. Finally, owing to the nature of pharmacists’ interventions in this study, participants and the investigators could not be blinded to the study intervention.

The initial reduction in BP by pharmacist-led interventions has important implications for primary and secondary prevention of cardiovascular morbidity and mortality. For example, evidence from a meta-analysis reported that even a 2-mmHg reduction in systolic BP could reduce the risk of stroke by 10% in the USA [10]. Another analysis suggests that a sustained 2-mmHg reduction in diastolic BP would be expected to result in a 6% reduction in the risk of coronary heart disease and 15% decrease in stroke [11]. However, since fewer participants were recruited in the study than required, findings of this study should therefore be interpreted with caution. Future studies with adequate sample size would be required to assess the effectiveness and sustainability of pharmacist-led interventions in the long term in clinical practice.

## Conclusion

This randomised controlled trial suggests that although written advice provided by community pharmacists in comparison to verbal advice was more effective in improving knowledge and understanding of patients about hypertension and its treatment, it did not lead to better blood pressure control.
